# Migration, breeding location, and seascape shape seabird assemblages in the northern Gulf of Mexico

**DOI:** 10.1371/journal.pone.0287316

**Published:** 2023-06-23

**Authors:** Pamela E. Michael, Kathy M. Hixson, Jeffery S. Gleason, J. Christopher Haney, Yvan G. Satgé, Patrick G. R. Jodice

**Affiliations:** 1 South Carolina Cooperative Fish & Wildlife Research Unit, Department of Forestry and Environmental Conservation, Clemson University, Clemson, South Carolina, United States of America; 2 U.S. Fish and Wildlife Service, Migratory Bird Program/Science Applications, Chiefland, Florida, United States of America; 3 Terra Mar Applied Sciences, Washington, District of Columbia, United States of America; 4 U.S. Geological Survey, South Carolina Cooperative Fish and Wildlife Research Unit, Clemson University, Clemson, South Carolina, United States of America; Texas A&M University, UNITED STATES

## Abstract

The Gulf of Mexico supports many seabird species, yet data gaps describing species composition and habitat use are prevalent. We used vessel-based observations from the Gulf of Mexico Marine Assessment Program for Protected Species to identify and characterize distinct seabird assemblages in the northern Gulf of Mexico (within the U.S. Exclusive Economic Zone; nGoM). Using cluster analysis of 17 seabird species, we identified assemblages based on seabird relative density. Vessel-based surveys documented the location, species, and number of seabirds across the nGoM between 2017–2019. For each assemblage, we identified the (co-)dominant species, spatial distribution, and areas of greater relative density. We also assessed the relationship of the total relative density within each assemblage with environmental, spatial, and temporal covariates. Of the species assessed, 76% (n = 13) breed predominantly outside the nGoM basin. We identified four seabird assemblages. Two assemblages, one dominated by black tern and the other co-dominated by northern gannet/laughing gull, occurred on the continental shelf. An assemblage dominated by sooty tern occurred along the continental slope into pelagic waters. The fourth assemblage had no dominant species, was broadly distributed, and was composed of observations with low relative density (‘singles’ assemblage). Differentiation of assemblages was linked to migratory patterns, residency, and breeding location. The spatial distributions and relationships of the black tern and northern gannet/laughing gull assemblages with environmental covariates indicate associations with river outflows and ports. The sooty tern assemblage overlapped an area prone to mesoscale feature formation. The singles assemblage may reflect commuting and dispersive behaviors. These findings highlight the importance of seasonal migrations and dynamic features across the seascape, shaping seabird assemblages. Considering the potential far-ranging effects of interactions with seabirds in the nGoM, awareness of these unique patterns and potential links with other fauna could inform future monitoring, research, restoration, offshore energy, and aquaculture development in this highly industrialized sea.

## Introduction

The northern Gulf of Mexico (which contains the Exclusive Economic Zone of the USA; nGoM) provides important habitat for a broad range of fauna [1, 2], one of the least studied components of which has been seabirds (but see [3]). This region supports a wide array of seabirds in terms of taxonomic diversity, geographic origin, foraging behavior, and conservation status [4]. For example, the breeding origins of seabirds that occur in the nGoM include six distinct geographic areas that span ˜120 degrees of latitude and both sides of the Atlantic; the northern Gulf coast, the continental interior of North America, the northeast coast of North America, the Caribbean, the eastern North Atlantic, and the western South Atlantic [4]. The unique annual cycles and migration patterns of species from each of these breeding origins result in a dynamic assemblage of seabirds, some of which occupy one, often spatially expansive, habitat type (e.g., pelagic or nearshore), while others occupy a broad range of habitats. For example, sooty terns (*Onychoprion fuscatus*), typically considered a pelagic tern, may also forage in estuarine and nearshore habitats [5]. Similarly, a species typically considered a nearshore occupant (e.g., laughing gull; *Leucophaeus atricilla*) might inhabit pelagic waters during specific life stages or times of year [6, 7]. The nGoM also presents a dynamic marine environment, occurring within both the Warm Temperate Northwest Atlantic and Tropical Northwestern Atlantic marine realms [8]. Despite being a semi-enclosed sea, the Gulf connects via multiple ocean currents to the Caribbean Sea and Gulf Stream, has complex bathymetry as well as substantial mixing of freshwater in estuaries, bays, and from river outflows [9]. This spatial and temporal variability in the seabirds present and the marine environment creates a mosaic of seabird species across the nGoM, with the potential for diverse drivers of occupancy and status.

Although previous survey efforts of seabirds in the nGoM have produced valuable assessments of species composition and habitat associations [1, 7], notable data gaps still exist. For example, previous surveys have often been restricted in spatial or temporal coverage [3, 6, 7, 10]. Some historical seabird surveys did not provide explicit details regarding either study design or data collection methods [1]. Surveys differed in the way seabird observations were described, where some recorded abundance while others only recorded presence, and not all surveys quantified the number of individuals observed at a given point in time and space [7, 10]. Some surveys intentionally excluded observations of certain species [6, 10]. These discrepancies in vessel-based seabird made it challenging, therefore, to fully ascertain the structure of the seabirds throughout the nGoM and the habitat or features they associate with.

There are numerous approaches that can be employed to describe the distribution of seabirds and their association with the environment. Each approach can provide novel insight into a different aspect of species-species and habitat associations and patterns. For example, hotspots in seabird abundance or species richness can be identified by overlapping the modeled distribution of individual species (e.g., [11]), while the relationship of individual species along environmental gradients can be identified through ordination techniques (e.g., [12]). Alternatively, simultaneously modeling the occurrence of multiple species through joint species distribution modeling can identify spatial structures or variability not captured by model covariates [13, 14]. As applied here, a seabird assemblage is composed of seabird species that display some shared characteristic or pattern (taxonomy, spatial distribution, co-occurrence, abundance) that is distinct from other assemblages. An assemblage does not require spatial proximity (although that may be a relevant factor) but rather can be defined across a range of factors. Furthermore, an assemblage simply implies a collection of species and does not imply direct interspecific interactions. Such interrelationships (with seabirds or with other marine fauna) would instead be defined as a community, implying functional relationships and mechanisms linked to seabirds. Here, we focus on identifying assemblages of seabirds.

Research on seabirds in systems ranging from tropical to polar, marginal sea shelf and open water environments has identified assemblages that are structured around spatiotemporal patterns and environmental factors. For example, seabirds often show a distinct onshore-offshore spatial pattern in abundance, where abundance follows a gradient across the continental shelf, continental slope, and pelagic habitats [15–17]. Temporal factors can also influence seabird assemblages and communities, whereby both species composition and abundance change among seasons (e.g., as species immigrate to or emigrate from marine regions [18, 19] or as a function of year (e.g., due to annual variation in large-scale oceanographic processes [18, 20, 21]. Breeding location can also strongly influence assemblage structure, with species that breed proximate to the study area often dominating the taxonomic composition of an assemblage or community [16, 22, 23]. Seabird assemblages and communities can also differ in their relationships with oceanographic processes and features such as ocean currents, thermocline depth, and saltwater-freshwater convergence zones where mixing occurs [23, 24]. Identifying seabird assemblages and determining the variables that structure them can help identify local and regional factors and processes that may influence these unique groups’ recurring or persistent formation.

Given the lack of survey and research attention on seabirds in the nGoM compared to many other marine regions and the potential vulnerability of seabirds there to a wide range of environmental and anthropogenic stressors (e.g., offshore energy production, heavy shipping traffic, potential aquaculture) [4, 25], we sought to identify and characterize the structure of distinct seabird assemblages in offshore waters. We used observations from vessel-based surveys collected as a part of the Gulf of Mexico Marine Assessment Program for Protected Species (GoMMAPPS), which have not previously been used to characterize seabird assemblages in the nGoM. Despite the distinct physical and oceanographic dynamics of the nGoM, we hypothesized that similar features and patterns would shape seabird assemblages in the nGoM as in other regions (e.g., onshore-offshore gradients, temporal variation, dynamic ocean features). We tested this hypothesis by identifying distinct seabird assemblages based on relative density (observations not adjusted for detectability) and characterizing their geographic location, species compositions, and areas of greater or lesser relative density. We then explored the association of each assemblage with environmental covariates that represented static, dynamic, temporal, and spatial factors. We interpreted these relationships with respect to the ecology of the most abundant species within each assemblage and regional oceanography. We also considered if the identified associations suggested links to other fauna, such as fish, that could infer community relationships. Our characterization of distinct seabird assemblages is the first of its kind for the nGoM and identifies important features shaping seabird assemblages, potentially informing future research, development, and monitoring in a region facing substantial pressure from both natural and anthropogenic stressors [4].

## Materials and methods

### Data sources

#### Seabird observations

**Fig 1:**
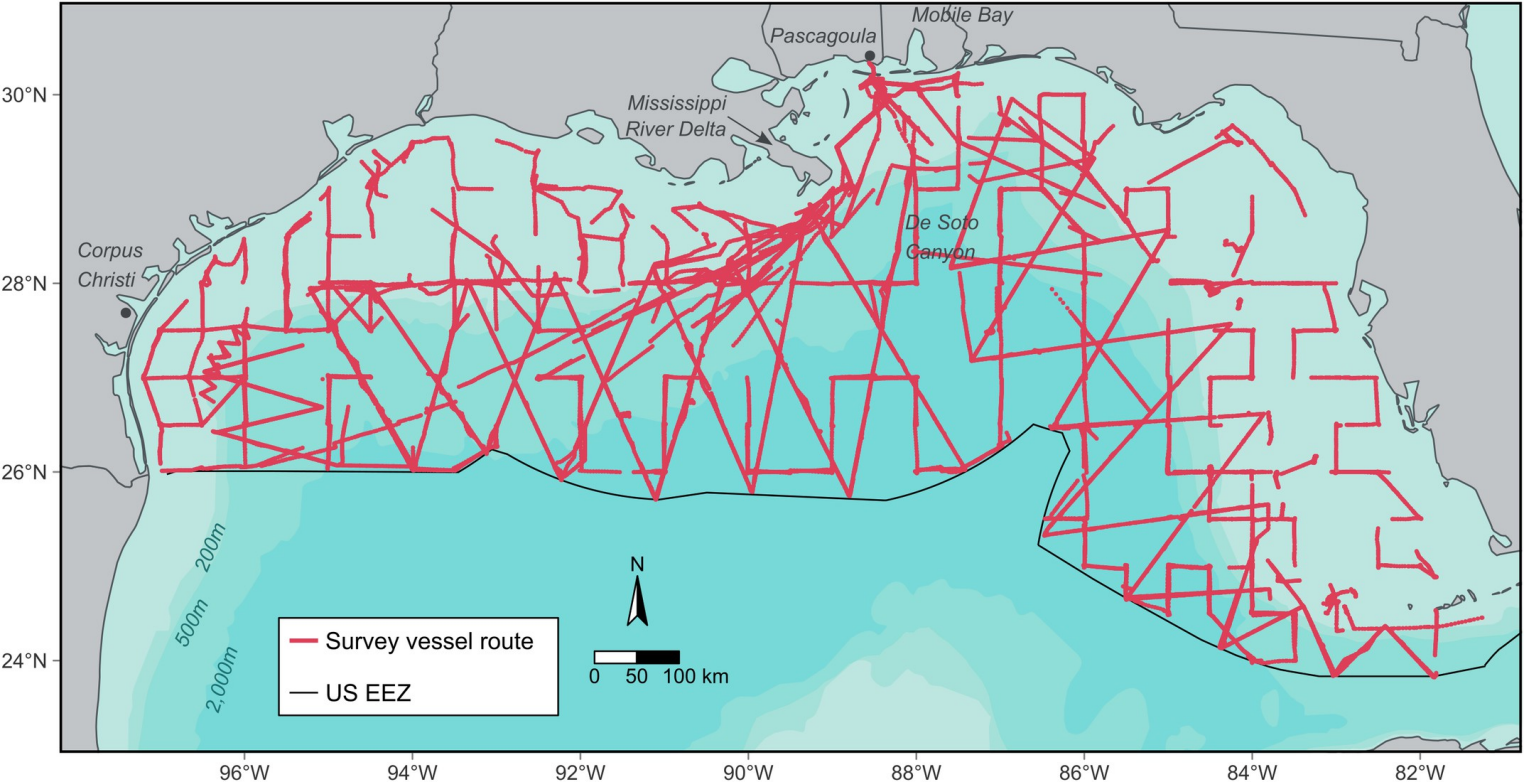
Study area and vessel survey footprint for vessel-based seabird observations for the Gulf of Mexico Marine Assessment Program for Protected Species (GOMMAPPS) program, 2017–2019. Red lines indicate the locations of vessel-based survey effort. The “U.S. EEZ” is the United States Exclusive Economic Zone.

We used seabird location and abundance data from vessel-based surveys collected as a part of the Gulf of Mexico Marine Assessment Program for Protected Species (GoMMAPPS). Data were collected on National Oceanic and Atmospheric Administration (NOAA) vessels in which track line placement, timing, and direction were pre-determined and designed specifically for conducting NOAA GoMMAPPS joint marine mammal and bird surveys. Data were also collected on ships of opportunity during NOAA programmatic surveys for fisheries and/or plankton data. Each survey type used a replicated survey design; thus, the vessel bird survey team had no ability to change or alter individual track lines. The data collected from these surveys define the study area and span the (1) continental shelf- ≤ 200 m, (2) continental slope 200 m– 2,000 m, and (3) pelagic: > 2,000 m bathymetric regions in the nGoM (Fig 1). Waters within the jurisdiction of individual U.S. Gulf coast states (< 3 nautical miles for Louisiana, Mississippi, and Alabama coasts and < 9 nautical miles from Texas and Florida coast) and areas too shallow for survey vessels to operate safely were not surveyed. Therefore, our data reflect what is generally regarded as the offshore waters of the nGoM.

Data were collected following standardized strip transect protocols for seabird observations from vessel-based surveys [26–28]. An observer placed on the flying bridge or bow of the vessel identified to the lowest taxonomic level and counted all birds within view. Only observations identified to species are used in this analysis. Observations were made from the side of the vessel with the least glare, and the distance of birds from the ship was estimated and grouped into bins as follows: from 0–100 m, 101–200 m, 201–300 m, and beyond 300 m. Low densities of birds compared to other regions coupled with good observation conditions in the nGoM (e.g., calm seas, high visibility) generally allowed species-specific identification and accurate counts to ˜ 500 m on both sides of the vessel. Such opportunities are not common in areas with higher densities of seabirds, greater abundances of pursuit diving seabirds, greater abundances of sitting seabirds, or in areas with generally poorer visibility, such as the north Atlantic, north Pacific, or central Pacific (e.g., [17, 29]), but do frequently occur in the nGoM (also see [30]). Seabird behavior was recorded (e.g., sitting, flying, foraging), and ship-following birds were identified and tracked to avoid counting the same individual multiple times [27]. Observations were made between sunrise to sunset but were suspended if conditions were deemed unsafe or the vessel had to go off survey effort for any reason, e.g., mechanical, weather, or other science tasks [27]. Data were collected from April 2017 –September 2019, with ˜ 290 days at sea representing ˜ 2,300 hours of observer effort for ˜ 41,700 total km of transects, with observations occurring in all months, excluding November and December. This represents the most extensive vessel-based survey effort for seabirds in the nGoM to date. A total of 44 seabird species from 12 taxonomic families were observed (S1 Table). Seabird vessel-based survey observation data can be accessed through the National Centers for Environmental Information (NCEI) archives: https://www.ncei.noaa.gov/archive/accession/0247206 and DOI https://doi.org/10.25921/afrq-h385 [31].

#### Environmental covariates

To describe the habitat of seabird assemblages in the nGoM, we selected five environmental covariates previously identified as relevant to seabird habitats in the Gulf of Mexico [28, 32] and the Gulf Stream in the western North Atlantic, the latter sharing some similarities with the nGoM with respect to marine fauna [33, 34]. Depth, indicating the bathymetric domain, was obtained from the SMRT30+ version 6.0 30 arc second dataset [35]. Monthly chlorophyll-*a* data from Modis Aqua 4 kilometers L3 SMI served as a proxy for the primary productivity [36]. Daily sea-surface temperature and sea-surface salinity (indicators of water mass; bodies of water with similar properties), and sea-surface height (indicating hydrographic features including convergence and divergence) were obtained from the Hybrid Coordinate Ocean Model (HYCOM; [37, 38]). All covariates are summarized in Table 1.

**Table 1:** Summary of the variables used in generalized additive models to assess the relationship between seabird relative density in each assemblage and environmental covariates. n/a = not applicable.

Covariate	Units	Ecological Context	Dataset name	Temporal Resolution	Data source	Associated references
Month^1^	n/a	Annual cycle, migration	n/a	daily	n/a	n/a
spatial surface	degrees longitude, latitude	Spatial structure, spatial autocorrelation	n/a	n/a	n/a	n/a
sea-surface temperature	degrees Celsius	Relates to water-mass	HYCOM	daily^2^	https://www.hycom.org/	[37, 38]
sea-surface salinity	practical salinity units	Relates to water-mass	HYCOM	daily^2^	https://www.hycom.org/	““
sea-surface height	Meters	Hydrographic features, convergence/divergence	HYCOM	daily^2^	https://www.hycom.org/	““
chlorophyll-*a*	milligrams / meter^3	Proxy for primary productivity	MODIS Aqua L3 CHLA Monthly 4km	monthly	http://apdrc.soest.hawaii.edu/datadoc/modis_aqua_chla.php	[36]
bathymetry	meters (above sea level)	Bathymetric domain	SMRT30+ version 6.0 30 arc second	n/a	https://coastwatch.pfeg.noaa.gov/erddap/griddap/usgsCeSrtm30v6.html	[35]

^1^Seasons were defined as spring (March-May), summer (June-August), fall (September-November), and winter (December-February) [34].

^2^Hourly 2019 data were downloaded at daily intervals

### Data filtering and aggregation

Survey data were filtered for vessel speed and interpolated into 10–15 minute temporal bins [33, 34, 39]. We were unable to correct seabird observations for detectability [40] and therefore refer to the resulting observations as relative abundance. Seabird relative abundance and environmental data were associated with the mid-point of each bin. To standardize the spatial resolution of the data, we aggregated the mid-point locations of each bin into a 10 x 10 km cell for each day with survey effort across the study area [41, 42]. A spatial resolution of 10 x 10 km was chosen as it has been found to minimize spatial autocorrelation in oceanographic variables [41], as well as considered to be a distance that a seabird in flight could be visually attracted to other seabirds in these subtropical marine habitats [43]. Because the number of kilometers surveyed in each 10–15-minute bin can differ due to ship speed, we divided the relative abundance of seabirds by the number of kilometers surveyed multiplied by the strip width (2 * 500 m). This produced relative seabirds/km^2^ (relative density) in each cell and day combination (hereafter ‘cell-day’).

When more than one bin occurred within a cell on the same day, we calculated the mean relative density. Observations were not uniformly distributed in space or time (S1 Fig). Uneven spatial or temporal coverage may result in a characterization of seabird assemblages that is more sensitive to variation in seabird composition in the frequently surveyed area or periods relative to other areas or periods with fewer observations. Variability in seabird composition in less surveyed areas may not be fully captured. Thus, the inference from our composition-based characterization of seabird assemblages is constrained by the spatial and temporal distribution of survey effort. By standardizing the data to a 10 x 10 km daily resolution, however, we can account for different levels of effort in each 10 x 10 km cell on a given day, thereby reducing the potential bias resulting from non-uniform spatial or temporal survey effort. This standardization also enables comparisons and maximizes the number of cell and day combinations included in the analysis.

After a preliminary assessment, we included any species of seabird that occurred in at least 1% of all cell-days. This threshold provided a taxonomically diverse suite of species (S1 Table) while limiting the number of potentially vagrant or rare species included. As our interest was in characterizing seabird assemblages and species co-occurrence, we further filtered cell-days to include only those when > 1 species was observed. These steps resulted in 17 species being included in the final data set and 27 species being excluded. These 17 species encompassed ˜ 99% of the relative seabird density of all 44 species detected.

### Analytical approach

#### Characterization of seabird assemblages

To identify and characterize distinct seabird assemblages in the nGoM, we performed a k-means cluster analysis on species-specific relative densities of each cell-day [44]. Clustering is a machine-learning method that partitions sample units into more similar clusters than sample units in a different cluster. Thus, cell-days with a more similar combination of species-specific relative densities group into a different cluster from cell-days with less similar species-specific relative densities. Each cluster is assumed to represent a different seabird assemblage. K-means cluster analysis applies an iterative algorithm that begins with an arbitrary location for the center of each cluster. Each sample unit (cell-day) is associated with the nearest center, forming a temporary cluster. A new center is assigned based on the gravitational center of all sample units in the temporary cluster, and the process is repeated until the selection criterion is met [45, 46]. The optimal number of clusters, constrained to be between 2 and 17, the number of species in the analysis was selected based on the Hartigan criterion, which minimizes the within-cluster sum of squares [44]. The number of clusters was identified using the *NbClust* package [45] and the final clusters were defined using the kmeans function in the *stats* package in base R [47] with 5,000 random sets of points used to identify the centers and a maximum of 10 iterations.

We transformed the mean relative density of each seabird species in each cell-day using natural log + 1. This monotonic transformation reduced the overall range in relative density values across species and maintained their rank order. The cluster analysis was run on the Euclidian distance matrix of the natural log + 1 transformed values. The clustering process did not involve geographic coordinates or temporal labels (e.g., ‘spring’, ‘April’). As clustering is based on seabird composition and observations of similar composition can occur across space, the breadth of the spatial footprint of an assemblage is not indicative of cluster cohesion. To assess the cohesion among observations in each cluster, we used the silhouette coefficient and visualization function in the *‘cluster’* and *‘factoextra’* packages in R, respectively [48, 49]. The silhouette coefficient is calculated by comparing the distance between points within a cluster to the distance to the nearest point in the neighboring cluster, where 1 indicates ideal clustering, -1 indicates observations are in the wrong cluster, and 0 indicates that observations are between two (unspecified) clusters (cluster is not highly distinct). A single species can occur in multiple clusters if cell-days with the species have sufficiently different species compositions to be assigned to different clusters by the clustering algorithm. We performed a single cluster analysis with all observations instead of season-specific analyses as 1) the definition of ‘season’ may not be appropriate or relevant for a given species (e.g., ‘boreal spring’ may differ phenologically for a northern versus southern Atlantic breeder), and 2) periods with low survey effort may have insufficient data to perform a distinct cluster analysis, resulting in their being excluded from analyses.

As multiple species can occur in an assemblage, we focused our interpretation on the individual species or combination of species summing to ≥ 50% of the total relative density within the assemblage; (hereafter ‘dominant’ or ‘co-dominant’, respectively). This ≥ 50% threshold is more conservative than > 25%, which has been used in other studies using a similar approach to characterize seabird assemblages [20, 23]. A ≥ 50% threshold provides a more intuitive interpretation of ‘dominant’ species than > 25%, but a smaller threshold may be more appropriate in regions where the number of species is greater than in the sub-tropics and tropics [20, 23]. In cases where just two species encompass 50% of the total relative density of that assemblage, the two species are described herein as ‘co-dominant’. To place each assemblage within the context of regional dynamics, we describe the overlap with regionally important environmental features, such as major river outflows and bathymetric domains, and identify areas of greater relative density.

*Association with environmental covariates*. Our second aim was to assess the relationship between the relative density of seabirds in each assemblage and environmental covariates (Table 1). This was done using generalized additive models (GAMs), a flexible modeling approach capturing non-linear relationships between environmental covariates [50]. We modeled each assemblage individually, using the sum of the relative densities of all seabirds in each cell within the assemblage as the response variable. In addition to the environmental covariates listed above (depth, chlorophyll-*a*, sea-surface temperature, sea-surface salinity, and sea-surface height), we also considered spatial and temporal covariates. To account for potential spatial-autocorrelation and spatial structure, we include a spatial smooth with an interaction between latitude and longitude in each model [51, 52]. This created a two-dimensional spatial surface. Measured distance to known breeding colonies or distance to shore have been used in studies describing the habitat associations of seabird assemblages [53]. However, including such a variable complicates analyses and interpretation when species breeding outside the study area are considered [15, 16]. Given our interest in the contribution of species breeding predominantly outside of the nGoM to the nGoM’s seabird assemblage and a strong correlation (Spearman’s rho: -0.871) between bathymetry and distance to land, we did not include distance to land in our analysis. In light of the occurrence of migratory seabirds in the nGoM [4] and the potential seasonal movements of non-migratory species, we included month as a covariate in the analysis.

We used the Tweedie distribution with a log link function to fit all models and applied thin-plate splines to all covariates, excluding the spatial smooth [54, 55]. Model fit diagnostics (q-q plots, residuals, Restricted Marginal Likelihood, Akaike’s Information Criterion, percent deviance explained) assuming Poisson, gamma, and log-gaussian distributions indicated a weaker model performance than models using a Tweedie distribution. The scale parameter was estimated during model fitting. We used the ‘choose.k’ function in the ‘*mgcv*’ package for R to select the number of dimensions for each spline [56]. If this produced an error, we constrained the number of knots in each environmental covariate modeled with a thin-plate spline to 1/10 of the cell-days for that assemblage. We used the restricted marginal likelihood (REML) for smoothness selection as generalized cross-validation (GCV), as GCV is prone to underestimating parameter values [57]. As we intended to create strong habitat models incorporating all of the above covariates, as opposed to the most parsimonious models, we included all covariates. We focus our discussion on each model’s statistically significant (p ≤ 0.05) variables. We also interpreted the relationship of relative seabird density in each cluster by assessing the smoothed curve of each covariate. All analyses were performed in R version 4.1.1, all functions related to GAMs were performed using the ‘*mgcv*’ package for R, and maps were created using the *‘tmap’* package for R [47, 56].

## Results

### Final dataset

**Fig 2:**
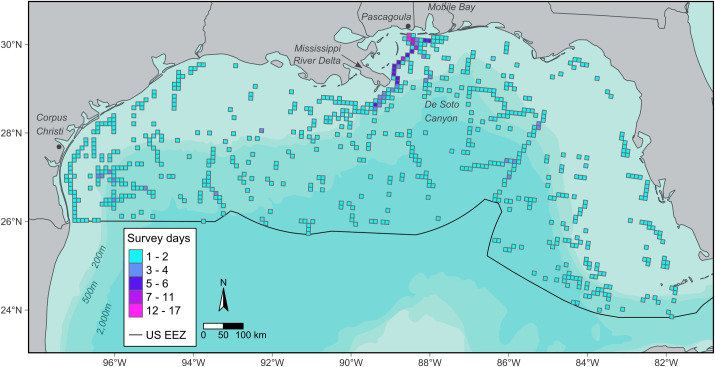
The number of days with observation effort in each 10 x 10 km cell was used to characterize seabird assemblages in the northern Gulf of Mexico. Darker shades indicate more days with observations. The “U.S. EEZ” is the United States Exclusive Economic Zone.

**Fig 3:**
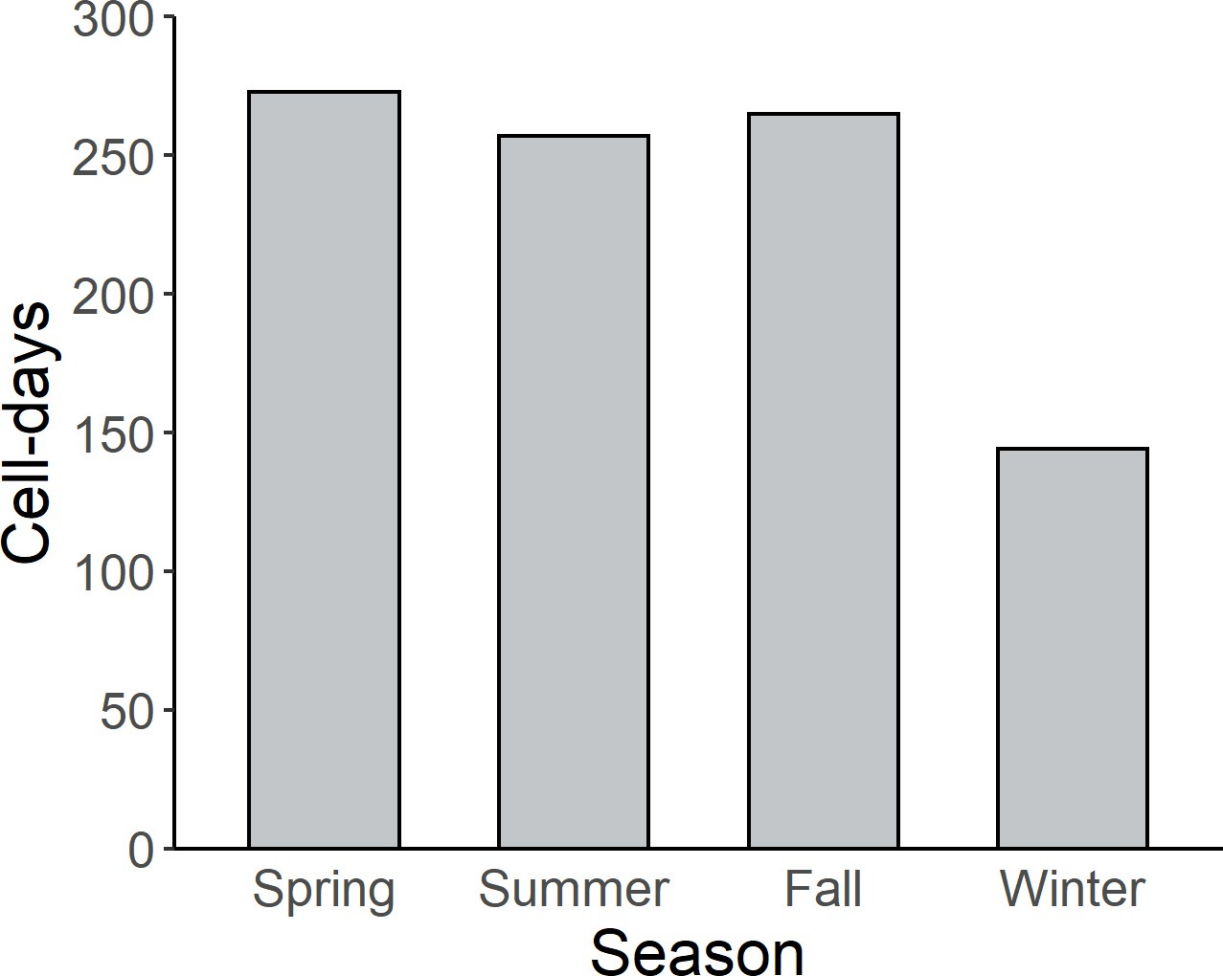
The total number of 10 x 10 km cell- days included in the characterization of seabird assemblages in the northern Gulf of Mexico, grouped by season. Seasons are defined as spring = March-May, summer = June-August, fall = September-November, and winter = December-February. Cell-day = a single 10 x 10 km cell with seabird observations in a given day.

Data filtering resulted in 939 unique cell and day combinations. Cells with observations occurred within all bathymetric domains and all portions of the study area, but the number of days with observations in a given cell was not distributed uniformly across the study area (Fig 2). Cells with multiple days of observation occurred near the port of Pascagoula (i.e., a common port of departure and return for most GoMMAPPS vessel surveys). Although data from all seasons are included in the analysis, seasonal coverage was uneven (Fig 3 and S2 Fig). Specifically, the number of cell-days included in the analysis was similar in spring, summer, and fall: 273, 257, and 265, respectively, and less in winter (144; S2 Fig).

The 17 species included in the analysis represent seven taxonomic families (Table 1). The species with the greatest maximum relative densities (individuals per km^2^) were northern gannet (*Morus bassanus*; 173), black tern (*Chlidonias niger*; 154), and sooty tern (93) (Table 2). Five species had maximum relative densities between 10 and 50 seabirds/km^2^, and nine had maximum relative densities < 10 seabirds/km^2^ (Table 2). Of the 17 focal species, 35% breed in the southern Gulf or Caribbean, 24% within the nGoM, 24% within the continental interior of North America, 12% from the eastern North Atlantic, and < 1% from the western North Atlantic coast (Table 2).

**Table 2:** Summary of relative density (seabirds/km^2^) and the number of cell-days occupied by each species. Relative density summaries were calculated only for cell-days where the species was present. As cell-days were filtered for co-occurrence (> 1 species), multiple species occurred within each cell-day. Species are listed in decreasing order of mean relative density. “Breeding origin” is a generalization of the breeding range for each species and reflects where most individuals observed in the nGoM are assumed to breed based on geographical ranges and migratory patterns. Cell-day = a single 10 x 10 km cell with seabird observations in a given day.

Common name	Family	Genus species	Breeding origin	Mean	Median	Minimum	Maximum	Standard deviation	# of cell-days	% of cell-days
Black Tern	*Laridae*	*Chlidonias niger*	Northern migrant–continental interior or high Arctic	5.8	0.7	0.0	153.8	15.8	202	21.5
Northern Gannet	*Sulidae*	*Morus bassanus*	Northern migrant–Atlantic coast	4.4	0.2	0.1	175.3	23.7	59	6.3
Sooty Tern	*Laridae*	*Onchyoprion fuscatus*	Southern Gulf, Caribbean	3.2	0.8	0.0	93.0	8.1	198	21.1
Sandwich Tern	*Laridae*	*Thalasseus sandvicensis*	nGoM	1.3	0.4	0.1	21.3	2.7	134	14.3
Herring Gull	*Laridae*	*Larus argentatus*	Northern migrant–continental interior or high Arctic	1.0	0.3	0.1	27.9	2.7	*175*	18.6
Laughing Gull	*Laridae*	*Leucophaeus atricilla*	nGoM	0.9	0.2	0.1	28.7	2.7	312	33.2
Brown Pelican	*Pelecanidae*	*Pelecanus occidentalis*	nGoM	0.7	0.2	0.1	8.1	1.3	112	11.9
Audubon’s Shearwater	*Procellariidae*	*Puffinus lherminieri*	Southern Gulf, Caribbean	0.6	0.2	0.0	19.8	2.1	192	20.4
Common Tern	*Laridae*	*Sterna hirundo*	Northern migrant–continental interior or high Arctic	0.5	0.2	0.1	3.0	0.7	63	6.7
Royal Tern	*Laridae*	*Thalasseus maximus*	nGoM	0.5	0.2	0.1	7.7	0.8	326	34.7
Pomarine Jaeger	*Stercorariidae*	*Stercorarius pomarinus*	Northern migrant–continental interior or high Arctic	0.5	0.2	0.1	12.6	1.3	124	13.2
Magnificent Frigatebird	*Fregatidae*	*Fregata magnificens*	Southern Gulf, Caribbean	0.5	0.2	0.0	6.7	0.8	179	19.1
Bridled Tern	*Laridae*	*Onchyoprion anaethetus*	Southern Gulf, Caribbean	0.3	0.1	0.1	4.3	0.5	108	11.5
Band-rumped Storm-petrel	*Hydrobatidae*	*Oceanodroma castro*	Eastern North Atlantic	0.3	0.2	0.1	2.5	0.3	109	11.6
Cory’s Shearwater	*Procellariidae*	*Calomectris diomedea*	Eastern North Atlantic	0.2	0.1	0.1	0.4	0.1	39	4.2
Brown Booby	*Sulidae*	*Sula leucogaster*	Southern Gulf, Caribbean	0.1	0.1	0.1	1.1	0.1	109	11.6
Masked Booby	*Sulidae*	*Sula dactylatra*	Southern Gulf, Caribbean	0.1	0.1	0.0	0.6	0.1	49	5.2

### Seabird assemblages

**Fig 4:**
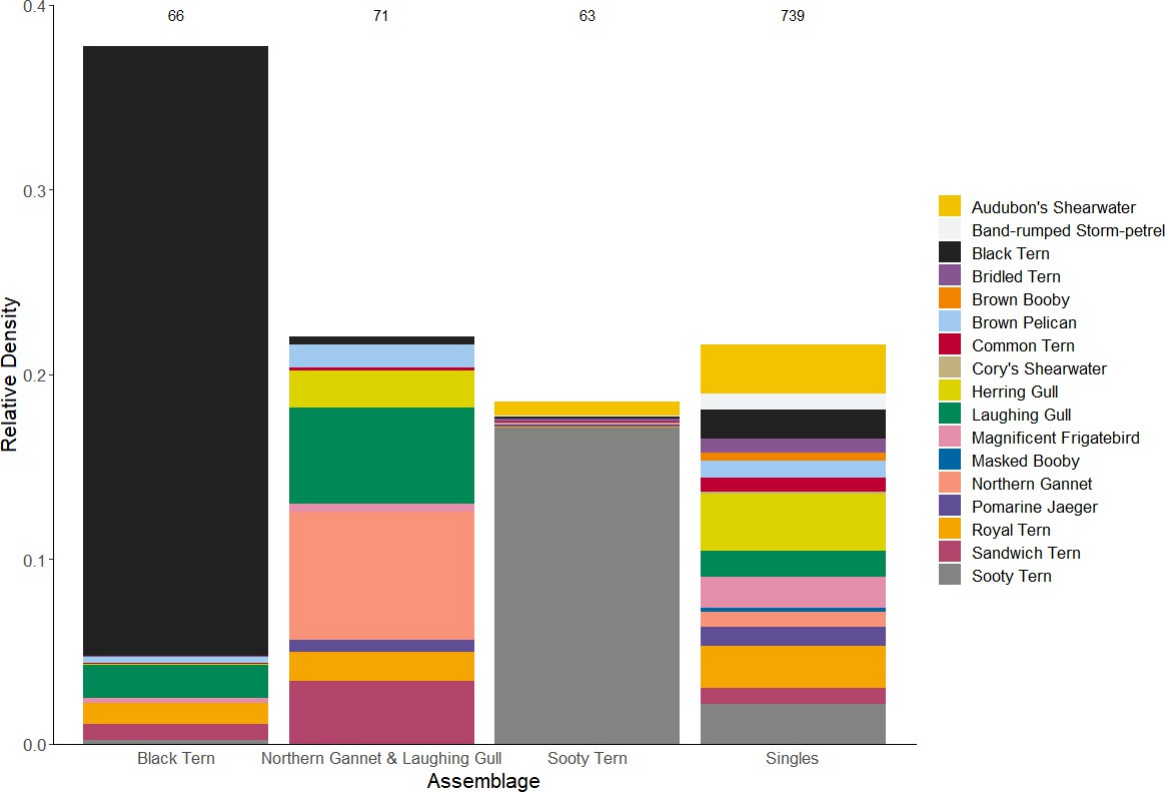
The total relative density, seabirds/km^2^, of each assemblage, by species. Assemblages were identified by k-means clustering of the data of seabird composition (co-occurrence and relative density). The number of cell-days in each assemblage is shown at the top of the column for each assemblage. The same species can occur in multiple assemblages. Bars for species occur in reverse alphabetical order of the common name from the bottom to the top of the stack. Cell-day = a single 10 x 10 km cell with seabird observations in a given day.

**Fig 5:**
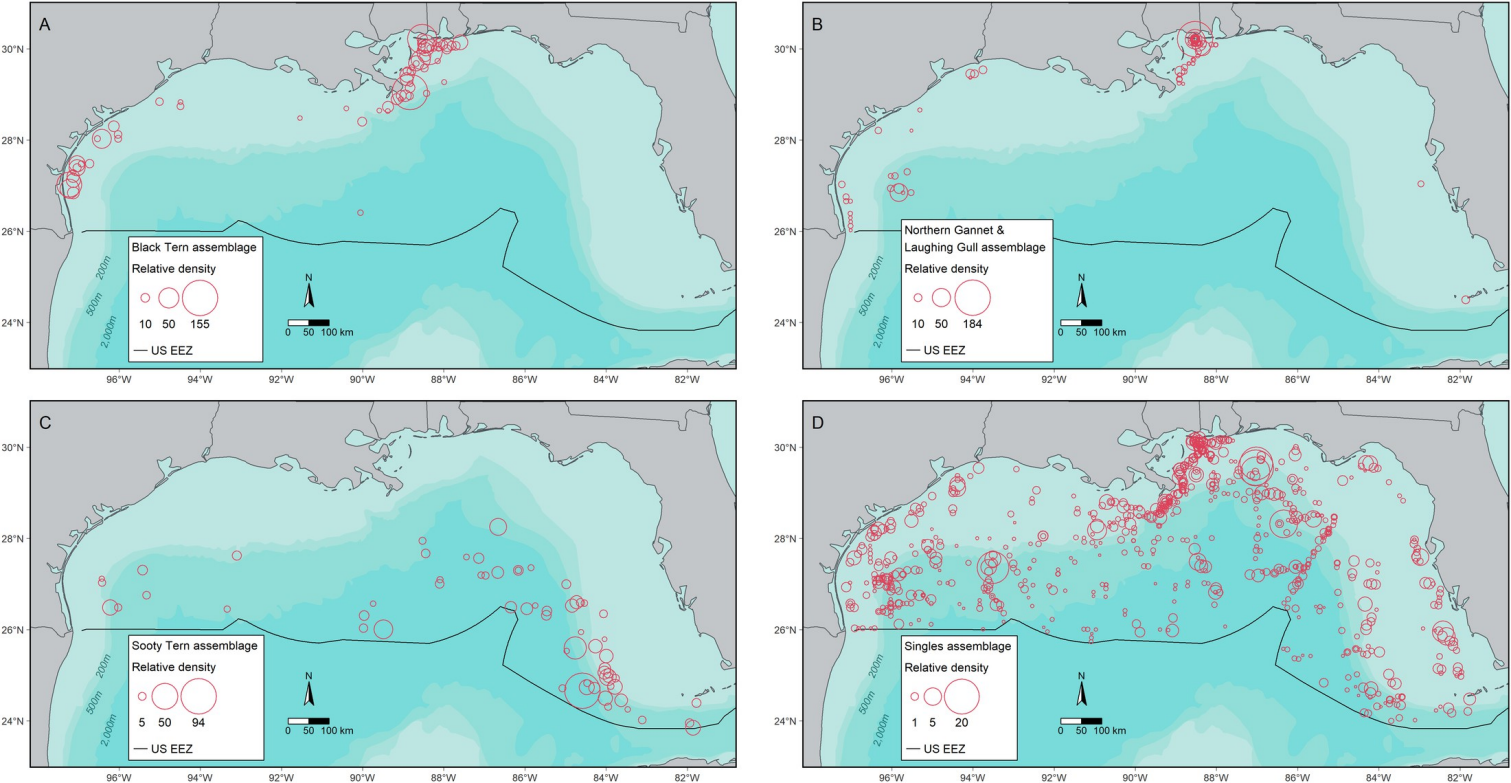
Spatial distribution and relative density, seabirds/km^2^, in each seabird assemblage. Assemblages are (A) black tern assemblage, (B) northern gannet/laughing gull assemblage, (C) sooty tern assemblage, and (D) singles assemblage. The radius of each circle reflects the relative density of seabirds, and the scale in each legend differs by assemblage. Multiple circles in one location indicate that observations were made in the same 10 x 10 km cell on different days. The “U.S. EEZ” is the United States Exclusive Economic Zone.

Three seabird assemblages were identified based on their dominant or co-dominant assemblage member(s). We defined these as (1) black tern, (2) northern gannet/laughing gull, and (3) sooty tern (Table 3 and Fig 4). A fourth assemblage lacked a dominant species and instead was characterized by the occurrence of many species with low relative densities. We refer to this assemblage as the ‘singles assemblage’ (Table 3 and Fig 4). Based on the silhouette coefficient, the cluster defining the singles assemblage had the highest goodness of fit (0.62), followed by the sooty tern assemblage (0.44) and the black tern assemblage, which had moderate measures of goodness of fit (0.3). The cluster defining the northern gannet/laughing gull assemblage had a silhouette coefficient of -0.12, indicating the cluster was not highly distinct (Table 3). The black tern and northern gannet/laughing gull assemblages occurred primarily on the western and eastern continental shelf (Fig 5A and 5B). The sooty tern assemblage was distributed along the continental slope (≥ 200 m– 2,000 m) in the central and eastern portions of the study area. In contrast, the singles assemblage was broadly distributed, including pelagic areas ≥ 2,000 m deep (Fig 5C and 5D).

**Table 3:** Summary of seabird assemblages. Dominant species were defined as those summing to ≥ 50% of species’ relative density for each assemblage. No dominant or co-dominant species are listed for the singles assemblage (Fig 4), as five species were needed to meet the ≥ 50% relative density threshold. Mean relative density relates to all cell-days within an assemblage. The silhouette coefficient assessed each cluster’s distinctness or ‘goodness’ where 1 indicates ideal clustering, 0 indicates that observations are between two clusters (cluster is not highly distinct), and -1 indicates observations are in the wrong cluster. Cell-day = a single 10 x 10 km cell with seabird observations in a given day.

Assemblage	(Co)-Dominant species (% relative density)	Mean relative density; seabirds/km^2^ (S.E.)	Sum of relative density; seabirds/km^2^	# of cell-days (% of cell-days in analyses)	Silhouette coefficient
Black Tern	Black Tern (87.5)	18.9 (3.16)	1,249	66 (7)	0.30
Northern Gannet/Laughing Gull	Northern Gannet (31.5), Laughing Gull (23.6)	10.3 (2.75)	729	71 (7.6)	-0.12
Sooty Tern	Sooty Tern (92.3)	9.73 (1.59)	613	63 (6.7)	0.44
Singles	No dominant species	0.968 (0.057)	715	739 (78.7)	0.62

In the black tern assemblage, black tern comprised ˜ 88% of the total relative density, and the six other species in this assemblage each contributed < 5% to the total relative density (Fig 4). This black tern assemblage had the greatest total and mean relative density (total = 1,249 and mean = 18.9 ± 3.16 SE seabirds/km^2^**)** of the four seabird assemblages we identified (Table 3 and Fig 4). This assemblage occurred primarily on the continental shelf shoreward of the 200 m isobath. Relative densities were greatest between the Mississippi River Delta and Mobile Bay, Alabama, and to a lesser extent, near Corpus Christi, Texas (< 200 m; Fig 5A).

In the northern gannet/laughing gull assemblage, northern gannet/laughing gull comprised 32% and 24% of assemblage-wide relative density, respectively (Table 3 and Fig 4). Eight other species were included within the assemblage, with four contributing 6–20% each to the assemblage. Four of the eight species, contributing a total of 30%, occur primarily in nearshore waters of the study area, and their occurrence is likely related to breeding and roosting locations throughout the study area: sandwich tern (*Thalasseus sandvicensis*, 15%), royal tern (*Thalasseus maximus*, 7%), brown pelican (*Pelecanus occidentalis*, 6%), and magnificent frigatebird (*Fregata magnificens*, 2%; Fig 4). The total (729 seabirds/km^2^) and mean (10.3 ± 2.75 seabirds/km^2^) relative density of the northern gannet/laughing gull assemblage was intermediate compared to the density of the other three assemblages (Table 3). This assemblage also occurred primarily on the continental shelf within the 200 m isobath. Peak relative densities for this assemblage occurred near Mobile Bay and consistently towards the Mississippi River Delta (Fig 5B). Moderate relative densities also occurred off Corpus Christi, Texas, at ˜ 200 m.

In the sooty tern assemblage, sooty tern comprised ˜ 92% of the total relative density (Table 3 and Fig 4). Five other species, each of which breeds outside of the Gulf, were included within this assemblage, although each contributed only ˜ 1–4% to the assemblage. The sooty tern assemblage had a mean relative density of 9.73 ± 1.59 SE seabirds/km^2^, similar to the northern gannet/laughing gull assemblage, but a lower total relative density, 613 seabirds/km^2^ (Table 3). The sooty tern assemblage occurred predominantly along the continental slope in the central and eastern portions of the study area (Fig 5C). The greatest densities for this assemblage occurred in the continental slope, 200 m– 2,000 m, off southwestern Florida.

The singles assemblage was unique in the lack of dominant/co-dominant species. This assemblage included all 17 species, and each contributed ≤ 15% to the assemblage (Fig 4). The five species that cumulatively contributed > 50% of seabird relative density included (in descending order), herring gull (*Larus argentatus*, 14%), Audubon’s shearwater (*Puffinus lherminieri*, 12%), royal tern (11%), sooty tern (10%), and magnificent frigatebird (8%) (Fig 4). The remaining 12 species contributed ≤ 7% each to the assemblage. This assemblage had the lowest mean relative density; 0.968 ± 0.057 SE seabirds/km^2^ but an intermediate total relative density of 715 seabirds/km^2^, which is similar to the northern gannet/laughing gull assemblage (Table 3). The singles assemblage was broadly distributed across the study area and had a notable presence in pelagic waters (Fig 5D). The greatest relative densities in this assemblage occurred near the DeSoto canyon, with moderate relative densities near the 200 m isobath down the continental slope (Fig 5D).

### Associations with environmental covariates

**Fig 6:**
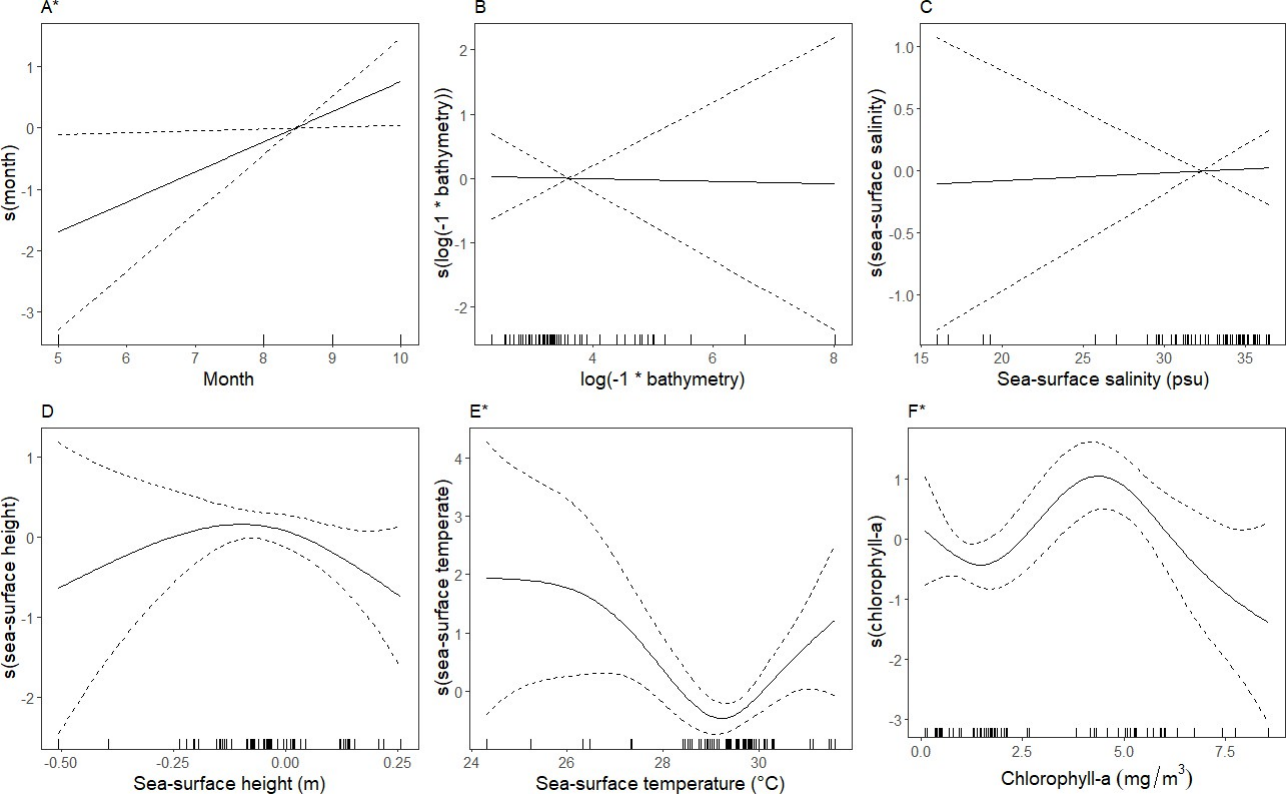
Smoothed curves of the additive effect of covariates on the estimated relative density of the black tern assemblage fitted with a generalized additive model. Dotted lines represent 95% confidence intervals, and each mark along the x-axis indicates a single observation at a given value. “*” indicates covariates that are significant at p ≤ 0.05. Tick marks on the x-axis (‘rug’) indicate the frequency each value of x was observed in the model.

**Fig 7:**
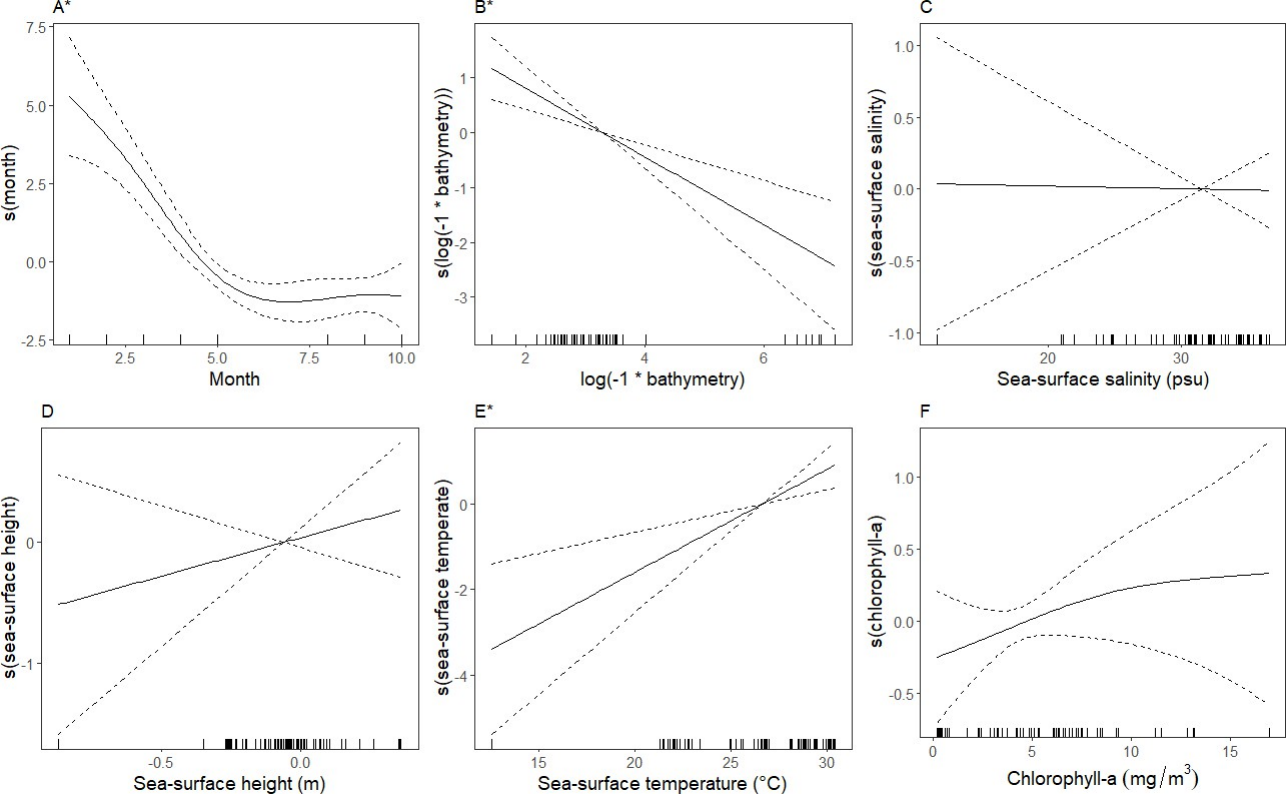
Smoothed curves of the additive effect of covariates on the estimated relative density of the northern gannet/laughing gull assemblage fitted with a generalized additive model. Dotted lines represent 95% confidence intervals, and each mark along the x-axis indicates a single observation at a given value. “*” indicates covariates that are significant at p ≤ 0.05. Tick marks on the x-axis (‘rug’) indicate the frequency each value of x was observed in the model.

Seabird assemblages differed in their relationships with environmental covariates. In the black tern assemblage, greater relative densities were significantly associated with date (September and October, boreal fall), and relatively high levels of chlorophyll-*a*, ˜ 5 milligrams/meter^3^ (Fig 6). This assemblage occurred primarily in waters with sea surface temperatures of ˜ 28 –˜31°C where greater relative densities occurred in warmer temperatures within this range. The model characterizing these relationships and those associated with the other environmental variables explained 52.0% of the deviance in the data (Table 4). In the northern gannet/laughing gull assemblage, greater relative densities were associated with date (January–May, boreal winter and spring) and relatively shallow bathymetry (Fig 7). This assemblage was observed in a patchy range of sea surface temperatures, with greater relative densities associated with temperatures, > 25°C. The model for the northern gannet/laughing gull assemblage had the highest percent of deviance explained (65.2%) of all four assemblages, indicating that the model captured a relatively high level of variability in the data (Table 4).

**Table 4:** Summary of generalized additive models assessing the relationship between seabird relative density in each assemblage with environmental covariates. Significant covariates are identified by a p-value ≤ 0.05 threshold.

Assemblage	Deviance explained	Significant covariates
Black Tern	52.0%	Month, temperature, chlorophyll-*a*
Northern Gannet/ Laughing Gull	65.2%	Month, depth, temperature
Sooty Tern	41.8%	Spatial surface, temperature
Singles	26.6%	Spatial surface, month, depth, sea-surface height,

In the sooty tern assemblage, greater relative densities were associated with areas with the interaction between longitude and latitude, indicating spatial autocorrelation and spatial structure in relative density patterns. Relative densities were also greater in sea-surface temperatures ˜ 25–29°C, decreasing with increasing temperature (Fig 8). The deviance explained for this assemblage was 41.8% (Table 4). In the singles assemblage, greater relative densities indicated spatial structuring and potential spatial autocorrelation through a significant relationship with the spatial surface (Table 4). Relative density was greater around March (boreal spring) and July–August (boreal summer) compared to other months (Fig 9). Greater relative densities were also associated with slightly low to neutral sea surface height; -0.2–0.0, and depths shallower than ˜500 m (Fig 9). The singles assemblage had the lowest percentage of deviance explained: 26.6% (Table 4).

**Fig 8:**
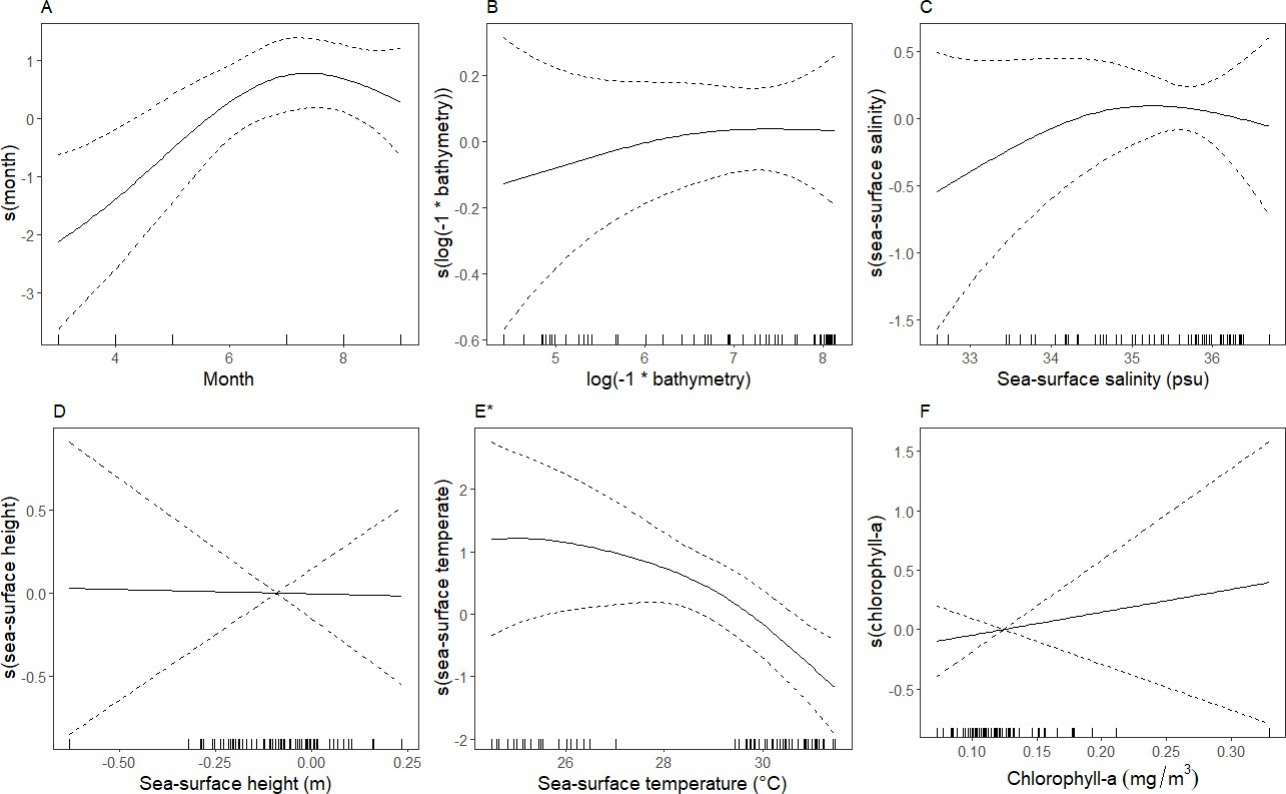
Smoothed curves of the additive effect of covariates on the estimated relative density of the sooty tern assemblage fitted with a generalized additive model. Dotted lines represent 95% confidence intervals, and each mark along the x-axis indicates a single observation at a given value. “*” indicates covariates that are significant at p ≤ 0.05. The spatial surface (interaction of longitude and latitude) was significant but is not shown. Tick marks on the x-axis (‘rug’) indicate the frequency each value of x was observed in the model.

**Fig 9:**
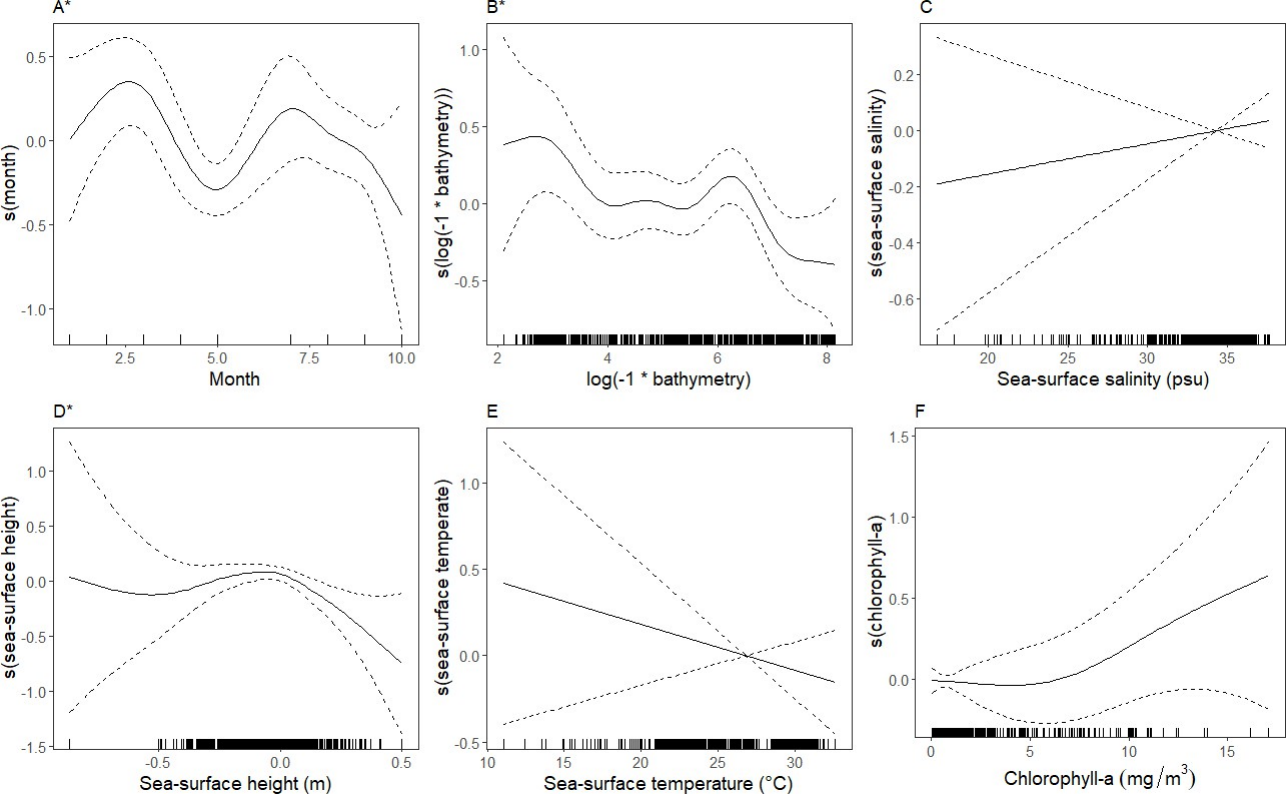
Smoothed curves of the additive effect of covariates on the estimated relative density of the singles assemblage fitted with a generalized additive model. Dotted lines represent 95% confidence intervals, and each mark along the x-axis indicates a single observation at a given value. “*” indicates covariates that are significant at p ≤ 0.05. The smoothed interaction between longitude and latitude (i.e., spatial surface) was also significant. The spatial surface (interaction of longitude and latitude) was significant but is not shown. Tick marks on the x-axis (‘rug’) indicate the frequency each value of x was observed in the model.

## Discussion

Using species-specific co-occurrence and relative density data from the most comprehensive dataset of seabird observations to date in the nGoM, we identified four broad-scale seabird assemblages in the region, each with a unique composition of species and a unique pattern in relative density both spatially and temporally. We found that features often associated with structuring seabird assemblages in other marine ecoregions were also relevant in the nGoM. For example, although the nGoM is a semi-enclosed warm temperate to tropical region with substantial freshwater mixing, we identified distinct seabird assemblages that were linked to unique seasonal patterns, environmental features, and breeding location, as has been found in previous research ranging from polar marginal seas, tropical and Antarctic open waters [16, 18, 21]. The consistency of these associations across studies in different marine regions underscores the roles that temporal variation (seasons, breeding cycles) and the seascape can play jointly in shaping offshore and pelagic seabird assemblages.

The high proportion of non-resident species in the assemblages we defined highlights the transboundary nature of seabird habitat use [58] and underscores that avifauna in the nGoM are not only a local or geographically isolated assemblage. For example, ˜ 76% of the species comprising the four assemblages we identified have breed predominantly outside of the nGoM, and 41% breed outside of the Gulf and the adjacent Caribbean region entirely. Of the four dominant species that were used to define our assemblages, three (black tern, northern gannet, and sooty tern) breed outside of the nGoM, although sooty tern also breed in the Dry Tortugas in the extreme southeastern corner of the study area. Migratory seabirds, therefore, play a major role in structuring the seabird assemblages of the northern Gulf. In some areas, locally breeding and migratory species differ in their feeding habitats and form unique assemblages [59–61]. Our findings indicate that, at a daily 10 x 10 km resolution, the habitats of probable residents compared to migratory species of seabirds in the nGoM are not always distinct (e.g., northern gannet/laughing gull assemblage). The structure of these assemblages demonstrates that, from a conservation perspective, environmental and anthropogenic stressors in the northern Gulf can have effects that extend across much of the north-south and east-west footprint of the Atlantic basin. As the understanding of the behavior of seabirds occurring in the nGoM increases through tracking (e.g., [62–65]), the connectivity of the nGoM to other regions will be revealed with increasing detail.

### Onshore-offshore gradient in relative density

Relative densities of seabirds in the nGoM were greater over the continental shelf than over the continental slope and in pelagic waters. A similar pattern of relative density with bathymetry has been observed in the Gulf Stream of the western North Atlantic, the southern Indian Ocean, and the eastern South Pacific [15–17]. During our study, the decrease in relative density with depth was driven by the greater average relative densities of the black tern assemblage and, to a lesser extent, the northern gannet/laughing gull assemblage, both of which occurred primarily on the continental shelf. In contrast, the sooty tern assemblage, primarily associated with the continental slope, had a slightly lower relative density. Similarly, the singles assemblage we defined had a much lower average relative density than the other assemblages and occurred primarily, although not exclusively, in pelagic waters. An array of dynamic features such as fronts, eddies, upwelling, and downwelling occur on the continental shelf and upper slope within the nGoM [66, 67]. These may provide enhanced foraging opportunities relative to pelagic areas, which in the nGoM tend to be broadly oligotrophic [68]. We discuss how each seabird assemblage may be associated with dynamic features associated with greater relative densities below.

### Assemblage-specific observations

#### Continental shelf assemblages

We identified two seabird assemblages with a strong affinity for shelf waters; one dominated by the migratory black tern and the other by the migratory northern gannet and resident laughing gull (although two other resident terns also occurred in this latter assemblage). These two assemblages had opposing trends in the relationship between season (months) and relative density. The black tern assemblage had greater relative density during the fall, while the northern gannet/laughing gull assemblage had greater relative density in the winter and spring. The difference appears to be due to differences in migration patterns between black tern and northern gannet, and residency patterns of laughing gull.

Black terns migrate from northern breeding grounds in the continental interior [69] in the fall when we observed them to be most numerous, and most birds continue to their wintering grounds in South America. In contrast, northern gannet typically depart from their breeding areas in northeastern Canada in late fall or early winter, with some birds overwintering along the Atlantic coast and others in the nGoM, before migrating back north in spring [70–72]. Historical records have also noted that northern gannet are more common in the northern Gulf in the winter than in other seasons [1, 7]. Regarding laughing gull, residents and occasional non-residents [73] may forage farther away from their coastal colonies and co-occur with northern gannet in the nGoM, once the gulls are no longer restricted to central place foraging during the breeding season. The same may be true for royal tern and sandwich tern. Although the composition of the northern gannet/laughing gull assemblage was not highly distinct, habitat models for all assemblages, including the black tern assemblage, indicated unique relationships with each seabird assemblage. The differentiation of the black tern and northern gannet/laughing gull seabird assemblages indicates that breeding and migratory dynamics may be an important factor shaping nGoM seabird assemblages (e.g., [18]) and that the temporal aspects of these dynamics are not identical among the geographically diverse breeding areas represented.

To different degrees and with different spatial patterns, greater relative densities of these two seabird assemblages associated with the continental shelf were observed near river outflows and the Mississippi River Delta, Mobile Bay, and Corpus Christi. These assemblages likely use additional river outflows and estuaries, but other river outflows and ports may not have been fully identified due to our survey coverage. In the black tern assemblage, the particularly high relative densities from the Mississippi River Delta to Mobile Bay, moderate relative density near Corpus Christi, and association with high chlorophyll-*a* indicate an association with river outflows or plumes. Large aggregations of seabirds in shelf areas influenced by river plumes were also observed by Louzao et al. [74] off the east coast of Spain and Zamon et al. [75] off the west coast of the United States. The association of seabirds with rivers could relate to the effect of nutrients introduced by rivers on the continental shelf. Specifically, river-derived nutrients can influence primary productivity [76] and support high rates of fisheries production [77]. Enhanced fish production could provide providing foraging opportunities for seabirds. Moreover, Zamon et al. [75] suggested that river plumes may serve as a mechanism for prey aggregation, similar to fronts in the pelagic environment. Combined with historical observations by Ribic et al. [7] and Davis et al. [1] of black tern near the Mississippi River Delta, interannual use of the Mississippi Delta region by black tern is highly likely. Black terns were observed foraging on large schools of bay anchovy (*Anchoa mitchilli*) during several GoMMAPPS surveys. Bay anchovy are the most abundant coastal fish in the western Atlantic Ocean, occupying riverine and marine environments and are tolerant of a broad range of temperatures and salinities [78]. Bay anchovy are an important component of the diets of breeding sandwich and royal tern in the region [79] and are also eaten by brown pelican [80]. The arrival of migrating black tern in the fall coincides with the general movement patterns of juvenile bay anchovy from brackish water in estuaries, bays, or river mouths to more saline, oceanic waters [81]. The nGoM could be an important staging area for black tern, providing a relatively reliable source of prey associated with river outflows in the fall on their migration to their wintering habitat.

Multiple, potentially interacting factors may shape the distribution of the northern gannet/laughing gull assemblage. Greater relative densities near Mobile Bay and moderate relative densities across the Mississippi River Delta align with observations made by Ribic et al. [7] and Haney [82], noting the tendency of northern gannet to forage near river outflows. The distribution of this assemblage also coincides with the distribution of Gulf menhaden (*Brevoortia patronus*), an important part of the diet of northern gannet and an important fishery in the region [83, 84]. Indeed, Montevecchi et al. [85] found that menhaden are an important part of the diets of northern gannet in the nGoM during winter. In addition to foraging directly on menhaden, northern gannet may forage on the discards from inshore fishing vessels. The moderate relative densities near fishing ports, including Corpus Christi and Mobile Bay, may relate to laughing gull also feeding on fisheries discards [28] or loafing near idle fishing boats. Fishing discards may be a common food source for this assemblage during the winter when productivity is generally lower and the relative density of the assemblage is greatest. Although not assessed, the moderate relative densities of this assemblage along the Gulf coast may be associated with the presence of laughing gull breeding areas in these areas.

#### Sooty tern assemblage

The distribution of the sooty tern- assemblage aligns with previous tracking data for the species from their breeding site in the Dry Tortugas. Sooty tern from this colony transit and forage on the continental slope off southwestern Florida, particularly in the area north of the Dry Tortugas, coinciding with high observed density for this seabird assemblage [62]. Along with a breeding colony of ˜40,000 pairs in the Dry Tortugas, other large colonies also occur in the southern Gulf (notably Campeche Bank), Bahamas, and the Caribbean [86]. When only considering the significant association of the sooty tern assemblage identified with sea surface temperatures 25–29°C in offshore waters, it is difficult to infer if sooty tern are associated with any particular marine feature. We posit that if this temperature range is considered in conjunction with regional oceanographic conditions along the eastern continental slope and central pelagic areas, this assemblage may co-occur with patchy mesoscale cold-core eddies, jets, or frontal features derived from the Loop Current [67]. An association of sooty terns with relatively cool dynamic features such as these could be related to the preferred habitat of tunas in the northern Gulf [87]. For example, sooty tern are known to have a near-obligate commensal relationship with tuna (e.g., [88]), where tuna drive prey to the surface and hence provide access for sooty terns to prey otherwise out-of-range (i.e., depth beyond the surface foraging capacity of sooty terns) in cold-core features [89].

Moreover, many of the sooty terns we observed were associated not only with tuna (*Thunnus spp*.*)* but also with Audubon’s shearwaters, the species with the second greatest relative density in the sooty tern assemblage [31]. These multi-species associations suggest this may be a functional community and not just an assemblage of co-occurring species. In the western Indian Ocean, Jaquemet et al. [90] found that sooty tern preferentially foraged in eddies, particularly in association with micronekton and tuna. The relationship between large flocks of sooty tern and tuna is documented well enough that fishers sometimes use flocks of sooty tern to locate schools of fish [91]. The potential association of sooty tern with dynamic features and direct observations co-occurring with tuna in the nGoM contributes to a growing body of literature suggesting that seabirds and seabird assemblages associate with mesoscale variation and features in the nGoM [32, 92].

#### Singles assemblage

The potential ecological associations of the singles community in the nGoM are challenging to identify, partially due to counterintuitive patterns. As spatial coordinates (latitude and longitude) were not a part of the cluster analysis, which was based on seabird composition, the breadth of the spatial distribution of a cluster (i.e., assemblage) does not reflect the distinctness of a seabird assemblage. The singles assemblage demonstrates this, as it has the broadest spatial distribution and is the most statistically distinct assemblage identified. The composition of this assemblage does not indicate strong co-occurrence patterns between specific members of the assemblage, nor does it capture large aggregations of individuals potentially foraging, engaged in area-restricted searching, or loafing as in other assemblages. This assemblage may instead reflect non-aggregation behavioral modes, such as commuting (migration or transiting between areas) or dispersive behavior of seabirds in the nGoM. Characterized by high velocity (e.g., [93]), these movement patterns could produce the diffuse distribution of observations assigned to the singles assemblage.

Many of the birds observed in the singles assemblage are non-residents. For example, four of the five species that comprise ˜ 50% of the relative density to this assemblage breed outside of the nGoM; Audubon’s shearwater, sooty tern, and magnificent frigatebird breed in the southern Gulf of Mexico or the Caribbean and herring gull breeds in the continental interior. Only royal tern breeds locally in the northern Gulf [4]. Recent vessel-based surveys in the tropical waters of French Guiana also observed high taxonomic diversity of seabirds, with species from local colonies and migrant species co-occurring in the same area [94]. The significant migratory component of the seabirds occurring in the nGoM may reflect the importance of sub-tropical and tropical waters to migratory and locally breeding species. Given the total relative density of this assemblage as well as its spatial and taxonomic breadth, a better understanding of the migratory behavior of seabirds using the nGoM could provide information guiding conservation or management efforts within and beyond the nGoM.

### Improving detection of environmental associations of seabird assemblages

The covariates used in our habitat models served as proxies for the features or conditions where seabirds may find productive foraging opportunities. Our understanding of the dynamics of seabird assemblages in the nGoM could be enhanced with data that provided a more direct connection to seabird diet, prey habitats and distribution, and features associated with foraging opportunities. Data characterizing the diet of seabirds in the nGoM could be used to understand the composition of seabird diet, the effects of diet, and the potential effects of environmental perturbation [80]. Information on the distribution of prey and other interacting species would allow for an explicit assessment of species co-occurrence [29, 95], a more accurate characterization of seabird distribution [96, 97], and a better understanding of predator-habitat relationships [98]. Standardized collection of data on *Sargassum*, a buoyant macroalgae that larval fish and other marine fauna associate with and can serve as a prey source for seabirds, has the potential to improve the understanding of how seabirds use habitats unique to the nGoM and interactions across taxa [99–101]. A significant challenge to characterizing habitat at such a broad scale is the potential mismatch of model resolution and pertinent environmental features. Specifically, our analysis’s 10 x 10 km resolution may not effectively capture the broad-scale oceanographic features potentially aggregating prey, such as the Loop Current. An enhanced understanding of the factors shaping and influencing this assemblage could help disentangle the potential versus probable relationships between species and species and nGoM habitats.

### Overlap with other megafauna

Many regions and features associated with high relative density of seabirds coincide with important habitats for other fauna. At broad spatial scales, much of the continental shelf areas used by the black tern and northern gannet/laughing gull assemblages are shared by delphinoids [102, 103], marine turtles, fish [104, 105], and potentially fish spawning aggregations [106]. The continental slope is used by many different cetaceans [107] and is strongly associated with dynamic features, including cold-core eddies and mesoscale features associated with locally concentrated zooplankton [108]. Covariate relationships suggest a potential link between the low relative density and sooty tern assemblages and dynamic habitats (e.g., low to neutral sea surface height in the singles assemblage). However, this study cannot confidently pinpoint explicit relationships between individual features and the seabird assemblage. Freshwater input near river outflows like the Mississippi and Atchafalaya and bays near Mobile and Corpus Christi are used by delphinoids [102]; snappers: [104], black-tip sharks [104], turtles [109–111], and menhaden [105]. The continental slope off southwestern Florida supports high relative densities of sooty tern, particularly during the breeding season [62], and could also support fish spawning aggregations [106]. The shared use of multiple habitats in the nGoM by a range of megafauna underscores the diversity and importance of nGoM habitats to multiple taxa. A more comprehensive investigation of multi-taxa use of nGoM habitats could better inform ecosystem-based management of marine taxa and the broader Gulf region, supporting the long-term priorities of the NOAA Resources and Ecosystems, Sustainability, Tourist Opportunities, and Revived Economies (RESTORE) program in the Gulf of Mexico.

## Conclusions

By characterizing seabird assemblages in the nGoM, we identified features affecting the distribution and abundance of the nGoM’s highly migratory and taxonomically diverse seabirds. Enhancing the understanding of an understudied component of the Gulf of Mexico Ecosystem (e.g., seabirds) supports the NOAA RESTORE Science Program’s mission of understanding the Gulf of Mexico Marine Ecosystem. Additional standardized observations at sea could track the distribution and composition of seabird assemblages over time. The resulting time series could be used to investigate the potential effects of environmental variation (e.g., [18, 20]), acute anthropogenic disturbance (e.g., [112]), and the long-term effects of vessel traffic. Such baselines could also inform the development of ongoing activities such as the siting and subsequent installation of offshore wind turbines and aquaculture facilities. Insight into migratory timing and connectivity of Gulf seabirds to other regions could be gained through tagging and tracking the movements of seabirds in the nGoM (e.g., [62, 63]). In addition to revealing the spatial extent of seabird habitat, seabird movement data could be used to better understand the factors affecting the occurrence, timing, and abundance of a given species and how that could affect species assemblages. Moreover, sustained seabird observations could be used in conjunction with observations in other locations along a species’ annual cycle, providing a more complete picture of the status and trends of seabirds occurring in the nGoM. The hypothesized links between distinct seabird assemblages and fish could be used to develop studies targeted toward establishing mechanistic links between seabirds and fisheries resources and support a comprehensive understanding of ecosystem services in the nGoM. With the anticipated diversification of anthropogenic activities in the nGoM (e.g., marine energy, culture and harvesting of marine species), standardized monitoring efforts, including pre-installation observations and assessments (e.g., [113, 114]), could improve insight into the effects of regionally novel offshore energy installation on the nGoM’s diverse seabird assemblages

## Supporting information

S1 Fig The total number of days surveyed in each 10 x 10 km cell by season in the northern Gulf of Mexico during the Gulf of Mexico Marine Assessment Program for Protected Species (GOMMAPPS) surveys, 2017, 2019. Seasons are defined as spring = March-May, summer = June-August, fall = September-November, and winter = December-February.
(DOCX)

S2 Fig The total number of days by season, with observations used in the characterization of seabird assemblages in the northern Gulf of Mexico. Seasons are defined as spring = March-May, summer = June-August, fall = September-November, and winter = December-February.
(DOCX)

S1 Table Summary of all seabird species observed in the northern Gulf of Mexico (nGoM) during Gulf of Mexico for the Gulf of Mexico Marine Assessment Program for Protected Species (GoMMAPPS) surveys, 2017–2019. "Breeding origin" is a generalization of the breeding range for each species and reflects where most individuals observed in the nGoM are assumed to breed based on geographical ranges and migratory patterns. "Detections "refer to a single observation; "individuals "refer to the number of individuals seen across all observations, "% individuals "compares the number of individuals observed for a given species to the total number of seabird individuals identified to species. Species in bold are included in the assemblage characterization analysis.
(DOCX)
