# Supplementary material for: Migration, breeding location, and seascape shape seabird assemblages in the northern Gulf of Mexico
Source: PLoS One. 2023 Jun 23;18(6):e0287316. doi: 10.1371/journal.pone.0287316 (PMC10289433; doi:10.1371/journal.pone.0287316)
Supplement: S2 Fig — Seasons are defined as spring = March-May, summer = June-August, fall = September-November, and winter = December-February. (DOCX) [file pone.0287316.s002.docx]

**Supporting information**


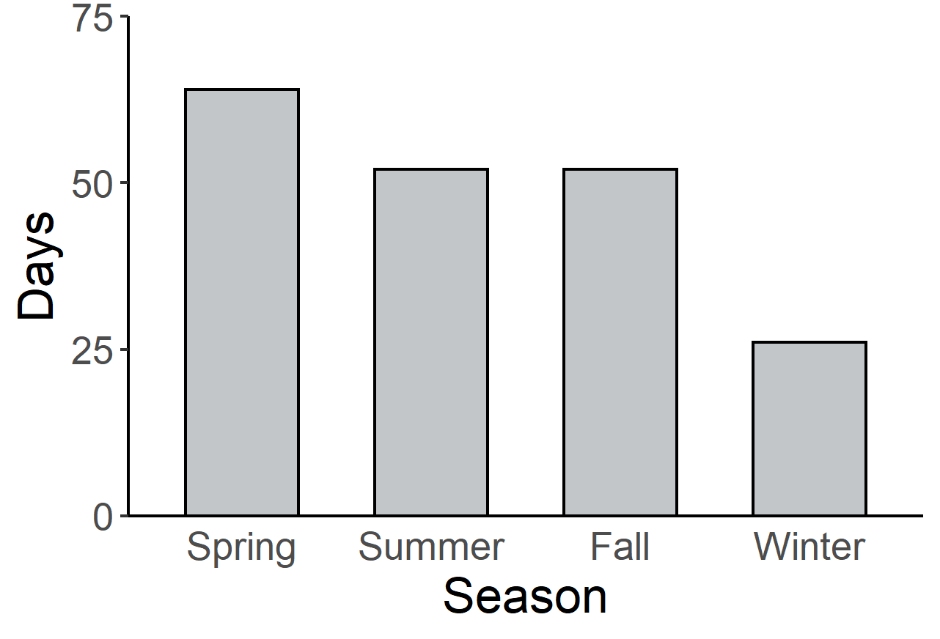


**S2 Fig.** **The total number of days by season, with observations used in the characterization of seabird assemblages in the northern Gulf of Mexico**. Seasons are defined as spring= March-May, summer= June-August, fall= September-November, and winter= December-February.
