# Supplementary material for: Migration, breeding location, and seascape shape seabird assemblages in the northern Gulf of Mexico
Source: PLoS One. 2023 Jun 23;18(6):e0287316. doi: 10.1371/journal.pone.0287316 (PMC10289433; doi:10.1371/journal.pone.0287316)
Supplement: S1 Table — "Breeding origin" is a generalization of the breeding range for each species and reflects where most individuals observed in the nGoM are assumed to breed based on geographical ranges and migratory patterns. "Detections "refer to a single observation; "individuals "refer to the number of individuals seen across all observations, "% individuals "compares the number of individuals observed for a given species to the total number of seabird individuals identified to species. Species in bold are included in the assemblage characterization analysis. (DOCX) [file pone.0287316.s003.docx]

**Supporting information**

**S1 Table. Summary of all seabird species observed in the northern Gulf of Mexico (nGoM) during Gulf of Mexico for the Gulf of Mexico Marine Assessment Program for Protected Species (GoMMAPPS) surveys, 2017-2019**. "Breeding origin" is a generalization of the breeding range for each species and reflects where most individuals observed in the nGoM are assumed to breed based on geographical ranges and migratory patterns. "Detections "refer to a single observation; "individuals "refer to the number of individuals seen across all observations, "% individuals "compares the number of individuals observed for a given species to the total number of seabird individuals identified to species. Species in bold are included in the assemblage characterization analysis.

| **Seabird category** | **Species** | **Family** | **Genus species** | **Breeding origin** | **Detections** | **Individuals** | **% seabirds** |
| --- | --- | --- | --- | --- | --- | --- | --- |
| Jaeger / Skua | South Polar Skua | *Stercorariidae* | *Stercorarius maccormicki* | South Atlantic | 1 | 1 | **0.00** |
| Cormorant | Neotropic Cormorant | *Phalacrocoracidae* | *Phalacrocorax brasilianus* | North GOM | 1 | 2 | **0.01** |
| Jaeger / Skua | Long-tailed Jaeger | *Stercorariidae* | *Stercorarius longicaudus* | Northern migrant - continental interior or high Arctic | 2 | 2 | **0.01** |
| Loon | Red-throated Loon | *Gaviidae* | *Gavia stellata* | Northern migrant - continental interior or high Arctic | 2 | 2 | **0.01** |
| Phalarope | Red Phalarope | *Scolopacidae* | *Phalaropus fulicarius* | Northern migrant - continental interior or high Arctic | 2 | 2 | **0.01** |
| Tern | Gull-billed Tern | *Laridae* | *Gelochelidon nilotica* | North GOM | 2 | 2 | **0.01** |
| Gadfly Petrel | Fea's Petrel | *Procellariidae* | *Pterodroma feae* | East Atlantic | 3 | 3 | **0.01** |
| Gull | Great black-backed gull | *Laridae* | *Larus marinus* | Northern migrant - Atlantic coast | 3 | 3 | **0.01** |
| Tropicbird | White-tailed Tropicbird | *Phaethontidae* | *Phaethon lepturus* | Southern Gulf / Caribbean | 3 | 3 | **0.01** |
| Shearwater | Manx Shearwater | *Procellariidae* | *Puffinus puffinus* | Northern migrant - Atlantic coast | 4 | 4 | **0.01** |
| Tern | Caspian Tern | *Laridae* | *Hydroprogne caspia* | North GOM | 6 | 7 | **0.02** |
| Gull | Ring-billed Gull | *Laridae* | *Larus delawarensis* | Northern migrant - continental interior or high Arctic | 8 | 9 | **0.02** |
| Booby | Red-footed Booby | *Sulidae* | *Sula sula* | Southern Gulf / Caribbean | 11 | 11 | **0.03** |
| Tropicbird | Red-billed Tropicbird | *Phaethontidae* | *Phaethon aethereus* | Southern Gulf / Caribbean | 12 | 12 | **0.03** |
| Phalarope | Red-necked Phalarope | *Scolopacidae* | *Phalaropus lobatus* | Northern migrant - continental interior or high Arctic | 10 | 16 | **0.04** |
| Tern | Least Tern | *Laridae* | *Sternula antillarum* | North GOM | 10 | 18 | **0.05** |
| Pelagic Tern | Roseate Tern | *Laridae* | *Sterna dougallii* | Southern Gulf / Caribbean | 5 | 20 | **0.05** |
| Storm petrel | Leach's Storm-Petrel | *Hydrobatidae* | *Oceanodroma leucorhoa* | Northern migrant - Atlantic coast | 19 | 24 | **0.06** |
| Gadfly Petrel | Black-capped Petrel | *Procellariidae* | *Pterodroma hasitata* | Southern Gulf / Caribbean | 29 | 31 | **0.08** |
| Storm petrel | Wilson's Storm-petrel | *Oceanitidae* | *Oceanites oceanicus* | South Atlantic | 27 | 34 | **0.09** |
| Shearwater | Great Shearwater | *Procellariidae* | *Ardenna gravis* | South Atlantic | 49 | 60 | **0.16** |
| Loon | Common Loon | *Gaviidae* | *Gavia immer* | Northern migrant - continental interior or high Arctic | 55 | 67 | **0.18** |
| Jaeger / Skua | Parasitic Jaeger | *Stercorariidae* | *Stercorarius parasiticus* | Northern migrant - continental interior or high Arctic | 43 | 73 | **0.19** |
| Tern | Forster's Gern | *Laridae* | *Sterna forsteri* | North GOM | 23 | 86 | **0.23** |
| **Shearwater** | **Cory's Shearwater** | *Procellariidae* | *Calonectris diomedea* | **East Atlantic** | **81** | **117** | **0.31** |
| Cormorant | Double-crested Cormorant | *Phalacrocoracidae* | *Phalacrocorax auritus* | North GOM | 33 | 130 | **0.34** |
| **Booby** | **Masked Booby** | *Sulidae* | *Sula dactylatra* | **Southern Gulf / Caribbean** | **124** | **136** | **0.36** |
| **Booby** | **Brown Booby** | *Sulidae* | *Sula leucogaster* | **Southern Gulf / Caribbean** | **300** | **355** | **0.94** |
| **Jaeger / Skua** | **Pomarine Jaeger** | *Stercorariidae* | *Stercorarius pomarinus* | **Northern migrant - continental interior or high Arctic** | **293** | **486** | **1.29** |
| **Tern** | **Common Tern** | *Laridae* | *Sterna hirundo* | **Northern migrant - continental interior or high Arctic** | **176** | **488** | **1.29** |
| **Pelagic Tern** | **Bridled Tern** | *Laridae* | *Onychoprion anaethetus* | **Southern Gulf / Caribbean** | **232** | **489** | **1.29** |
| **Storm petrel** | **Band-rumped Storm-petrel** | *Hydrobatidae* | *Oceanodroma castro* | **East Atlantic** | **334** | **512** | **1.35** |
| Pelagic Tern | Brown Noddy | *Laridae* | *Anous stolidus* | Southern Gulf / Caribbean | 117 | 595 | **1.57** |
| **Pelican** | **Brown Pelican** | *Pelecanidae* | *Pelecanus occidentalis* | **North GOM** | **240** | **814** | **2.15** |
| **Frigatebird** | **Magnificent Frigatebird** | *Fregatidae* | *Fregata magnificens* | **Southern Gulf / Caribbean** | **478** | **940** | **2.49** |
| Gull | Bonaparte's Gull | *Laridae* | *Chroicocephalus philadelphia* | Northern migrant - continental interior or high Arctic | 83 | 1356 | **3.59** |
| **Tern** | **Sandwich Tern** | *Laridae* | *Onychoprion fuscatus* | **North GOM** | **372** | **1445** | **3.82** |
| **Gull** | **Herring Gull** | *Laridae* | *Larus argentatus* | **Northern migrant - continental interior or high Arctic** | **856** | **1636** | **4.33** |
| **Gannet** | **Northern Gannet** | *Sulidae* | *Morus bassanus* | **Northern migrant - Atlantic coast** | **320** | **1658** | **4.38** |
| **Shearwater** | **Audubon's Shearwater** | *Procellariidae* | *Puffinus iherminieri* | **Southern Gulf / Caribbean** | **517** | **1766** | **4.67** |
| **Tern** | **Royal Tern** | *Laridae* | *Thalasseus maximus* | **North GOM** | **1104** | **1869** | **4.94** |
| **Gull** | **Laughing Gull** | *Laridae* | *Leucophaeus atricilla* | **North GOM** | **1086** | **2569** | **6.79** |
| **Pelagic Tern** | **Sooty Tern** | *Laridae* | *Onychoprion fuscatus* | **Southern Gulf / Caribbean** | **851** | **7855** | **20.77** |
| **Tern** | **Black Tern** | *Laridae* | *Chlidonias niger* | **Northern migrant - continental interior or high Arctic** | **726** | **12109** | **32.02** |
|  | *Total* |  |  |  | *8653* | *37817* |  |
